# Identification of Metabolites Changes and Quality in Strawberry Fruit: Effect of Cultivation in High Tunnel and Open Field

**DOI:** 10.3390/plants11101368

**Published:** 2022-05-21

**Authors:** Mustafa Kenan Gecer, Erdal Orman, Muttalip Gundogdu, Sezai Ercisli, Rohini Karunakaran

**Affiliations:** 1 Department of Seed Science and Technology, Agricultural Faculty, Bolu Abant Izzet Baysal University, 14030 Bolu, Turkey; mkenangecer@hotmail.com; 2 Ataturk Horticultural Central Research Institute, 77100 Yalova, Turkey; e.orman77@gmail.com; 3 Department of Horticulture, Agricultural Faculty, Bolu Abant Izzet Baysal University, 14030 Bolu, Turkey; gundogdumuttalip@gmail.com; 4 Department of Horticulture, Agricultural Faculty, Ataturk University, 25240 Erzurum, Turkey; sercisli@gmail.com; 5 Unit of Biochemistry, Centre of Excellence for Biomaterials Engineering, Faculty of Medicine, AIMST University, Semeling, Bedong 08100, Malaysia; 6 Department of Computational Biology, Institute of Bioinformatics, Saveetha School of Engineering (SSE), SIMATS, Thandalam, Chennai 602105, Tamil Nadu, India; 7 Centre of Excellence for Biomaterials Science, AIMST University, Semeling, Bedong 08100, Malaysia

**Keywords:** strawberry, high plastic tunnel, fruit characteristics, phenolic and organic acids

## Abstract

Strawberry has gained increasing popularity all over the world due to its fruit properties. This popularity is due to the phytochemicals that strawberries have. The aim of this study is to reveal the effect of cultivation in open field and high tunnel on agromorphological and biochemical properties of strawberries. In this study, fruit yield and fruit quality characteristics of some strawberry varieties grown in open field and high tunnel were investigated. The highest fruit yield, fruit weight and titratable acidity were obtained in high tunnel grown Albion cultivar (542.743 g/plant, 14.927 g/fruit and 1.047%, respectively). While there was no statistical difference between cultivars and treatments in terms of soluble solids content and pH, fruit count was higher in Albion and Kabarla cultivars in both treatments compared to other cultivars (*p* < 0.05). Among the phenolic compounds, gallic acid was determined to be higher (between 9.246–31.680 mg/100 g) than other phenolics. Considering the organic acid content, malic acid was determined as the dominant organic acid in Kabarla cultivar (870.729 mg/100 g). In addition, in terms of vitamin C content, Rubygem cultivar stood out in both applications. Phenolic compound and organic acid contents varied in terms of varieties and applications. As a result, strawberry fruit, which is an important fruit in terms of fruit quality and consumption diversity, has been found to have high phenolic compounds and organic acid content, although it varies in all varieties and applications.

## 1. Introduction

Strawberry is one of the most important fruit species in the *Fragaria* genus of the *Rosaceae* family, its cultivation is increasing worldwide, and in addition to its positive contributions to health, it is also one of the most important fruit species in terms of economic value [1]. Strawberry has recently become one of the most loved fruits all over the world. Strawberry is one of the most preferred fruit types among other berry fruits due to its color, high aroma, flavor, and bioactive compounds. Strawberry fruit is rich in bioactive compounds such as phenolic acids, flavonoids, sugars, organic acids, and anthocyanins [2]. Strawberry fruit, which is rich in biochemical compounds, is preferred by people who want a healthy diet [3]. Strawberry fruit is widely grown in many countries today and is preferred both for fresh consumption and as a functional product in many fields, especially in the food industry. As studies reveal the characteristics of this fruit species, its popularity is gradually increasing.

It has been reported that mulch application in strawberry cultivation increases the soil temperature and thus the yield increases [4]. By using greenhouse cultivation systems against adverse climatic conditions, it is possible to eliminate the problems that may occur in yield and quality increase. The use of greenhouse systems in strawberry production helps to protect plants from adverse environmental conditions and to spread the production over a long period of time, helping the producers to earn more income [5,6,7]. There are great differences in fruit quality of strawberry varieties grown in plastic tunnels or in the open field [8]. Significant differences were found among cultivars in terms of quality characteristics such as yield, fruit size, fruit color, taste, shape, ripening time, resistance to diseases etc. [9]. Due to these differences, regular and high-quality products are purchased throughout the production season in the world. Fruit size, fruit shape, amount of water-soluble dry matter, sugar content, acidity, taste, and aroma are important characteristics of strawberry fruit [10]. Bioactive compounds in the fruit show positive effects on human health due to their antioxidant, antiallergic, anticarcinogenic and antimicrobial effects. Studies have shown that the bioactive content in the fruit is largely dependent on the strawberry variety as well as the effect of seasonal changes [11,12].

Many studies have demonstrated the protective roles of phenolic compounds against coronary heart disease, stroke, and some forms of cancer. These protective effects of phenolic compounds are due to their antiradical activities in cells [13]. According to the results of different studies, it was determined that strawberry fruit contains high amounts of vitamin C [14] and phenolic compounds when compared to other fruits and vegetables [15,16,17,18]. In addition, because of its deliciousness, strawberry fruit, which provides the opportunity to sell at high prices because of the increase in consumer demand, especially in the spring, increases the interest in this fruit type because of its high mineral content as well as vitamin C [1,19].

There are many studies to determine the anticarcinogenic effect of anthocyanins, which give the strawberry fruit its red color, thanks to its health benefits [2,20]. Growing different strawberry varieties and genotypes [21] under different growing conditions and systems (covered with black polyethylene mulch and without mulch) affects their antioxidant content [22]. In addition, the high amount of ascorbic acid in strawberries provides protective properties on reactive oxygen radicals and reduces oxidative stress [23]. Strawberry fruit has been reported to prevent cancer in terms of ellagic acid and high antioxidant capacity [24]. Strawberry emerges as an important fruit in the Mediterranean diet because it contains essential nutrients and some beneficial phytochemicals that are effective on human health [3].

In this study, the effects of different cultivation techniques (open field and high plastic tunnel) on agromorphological properties, phenolic compounds, and organic acid contents of fruits of commercial strawberry varieties widely grown in Turkey were investigated. Cultural practices, genetic factors and ecological conditions are of great importance in fruit growing. These factors affect the quality characteristics and biochemical contents of fruits and determine their marketability. In high tunnel cultivation, earliness in particular leads to commercially selling fruits at higher prices and earning more money for the farmers. Therefore, although earliness is a known fact in high tunnel cultivation, it has been observed that the information on how organic acids and phenolic compounds change is limited. These metabolites are compounds with antioxidant properties that play an important role in human health and nutrition. Accordingly, in this study, strawberry varieties grown in high tunnel and open field were compared in terms of agromorphological quality characteristics and biochemical contents and it was revealed which cultivation is suitable.

## 2. Materials and Methods

This study was planned and carried out for two years between 2018–2019 in the ecological conditions of Iğdır province (Turkey). In the study, frigo seedlings of Albion, Kabarla and Rubygem strawberry cultivars were used in open field and high tunnel conditions. The seedling planting pads were covered with plastic mulch (black, 8 micron) after the drip irrigation pipe was placed, and the seedlings were planted on 12 May 2018 by cross planting method at 35 × 35 cm intervals. In this study, the analyzes were made on fresh fruit samples (fw).

### 2.1. Morphological Properties

For each application, the fruits were harvested and weighed on a 0.1 g sensitive balance, and the yield was determined by dividing the total yield per plot by the number of plants. The average fruit weight was determined by dividing the yield amount by the number of fruit. The number of fruits per plant was calculated by counting the harvested fruits and dividing them by the number of plants in the plot. Ten fruits selected by chance were crushed and juiced, and the amount of soluble solids contents (SSC) was determined with the help of a digital refractometer (Model HI-96801 Hanna GmbH, Wöhringen, Germany). Ten randomly picked fruits were crushed and juiced, and the electrode tip of the pH meter (Hanna-HI 98103, Hanna GmbH, Wöhringen, Germany) was placed in the juice. When the value displayed on the screen becomes stable, it was saved. It was calculated in terms of citric acid by titration technique in 10 mL fruit juice obtained from random fruits.

### 2.2. Determination of Phenolic Compounds

**Figure 1:**
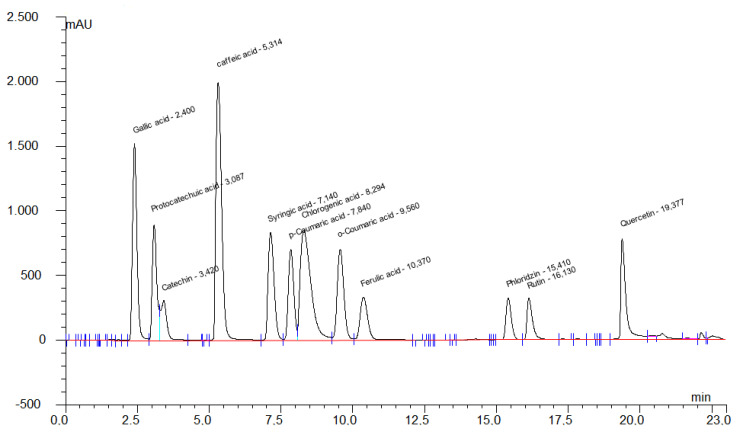
Chromatogram of phenolic compound standards.

In the study, phenolic compounds; gallic acid, protocatechuic acid, catechin, chlorogenic acid, caffeic acid, syringic acid, ferulic acid, *p*-coumaric acid, o-coumaric acid, rutin, phloridzin and quercetin (Merc, Germany) were determined. After 5 g of strawberry sample was crushed in a homogenizer, it is diluted with distilled water at a ratio of 1:1 and left for 15 min. It was centrifuged at 15,000 rpm. Then the supernatant was filtered with 0.45 μm millipore filters and injected into HPLC. Chromatographic separation was performed on Agilent 1100 HPLC system, using a 250*4.6 mm, 4 μm ODS column with a DAD detector (Figure 1). Solvent A Methanol-acetic acid-water (10:2:88), Solvent B Methanol-acetic acid-water (90:2:8) was used as mobile phase. Separation was performed at 254 and 280 nm and the flow rate were determined as 1 mL/min and the injection volume was 20 µL [25].

### 2.3. Organic Acid Determination

**Figure 2:**
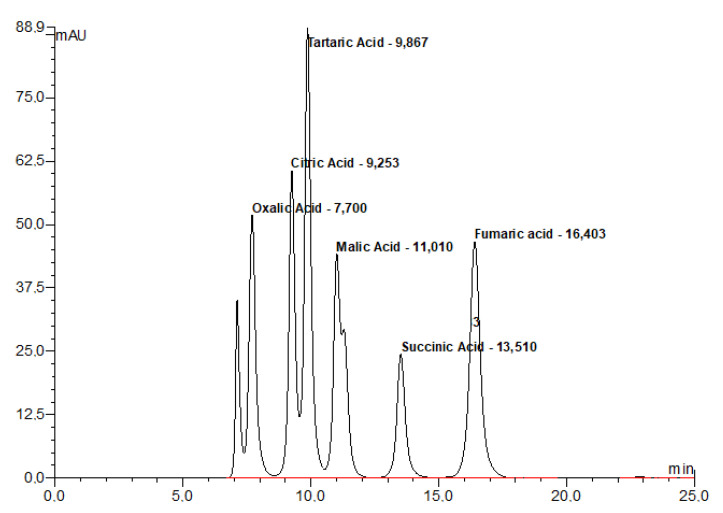
Chromatogram of organic acid standards.

The method of Bevilacqua and Califano [26] was modified and used in the extraction of organic acids. 5 g of the obtained fruit samples were taken and transferred to centrifuge tubes. These samples were homogenized by adding 20 mL of 0.009 N H_2_SO_4_ (Heidolph Silent Crusher M, Schwabach, Germany). Then it was stirred for 1 h on a shaker (Heidolph Unimax 1010, Germany) and centrifuged at 15,000 rpm for 15 min. The aqueous fraction separated in the centrifuge was first passed through coarse filter paper, then twice through a 0.45 μm membrane filter (Millipore Millex-HV Hydrophilic PVDF, Millipore, Burlington, MA, USA) and finally through the SEP-PAK C18 cartridge. Organic acids were analyzed in a high-performance liquid chromatography (HPLC) instrument (Agilent HPLC 1100 series G 1322 A, Germany) using the method specified by Bevilacqua and Califano [26]. Aminex HPX -87 H, 300 mm × 7.8 mm column (Bio-Rad Laboratories, Richmond, CA, USA) was used in the HPLC system and the device was controlled by a computer with Agilent package program (Figure 2). The DAD detector in the system (Agilent, USA) is tuned to 214 and 280 nm wavelengths. In the study, 0.009 N H_2_SO_4_ passed through a 0.45 μm membrane filter was used as the mobile phase.

### 2.4. Determination of Vitamin C

Strawberry fruit sample (15 g) was transferred to the test tube and 5 mL of 2.5% M-phosphoric acid solution was added. The mixture was centrifuged at 6500× *g* for 10 min at +4 °C. 0.5 mL was taken from the clear part in the centrifuge tube and made up to 10 mL with 2.5% M-phosphoric solution. This mixture was passed through a 0.45 μm Teflon filter and injected into the HPLC device. HPLC analyzes were performed on C18 column (Phenomenex Luna C18, 250 × 4.60 mm, 5 μm). The column furnace temperature was set at 25 °C. Ultrapure water with pH level adjusted to 2.2 with H_2_SO_4_ at a flow rate of 1 mL/min was used as the mobile phase in the system. Readings were made in a DAD detector at a wavelength of 254 nm. L–ascorbic acid (Sigma A5960, Saint Louis, MO, USA) prepared at different concentrations was used to identify and quantify the vitamin C peak [27].

### 2.5. Statistical Analysis

As a result of the study, which was carried out with 3 replications and 20 plants in each replication, the data obtained with two-year averages were subjected to analysis of variance based on the factorial trial design in random blocks. Duncan’s multiple comparison test was used to identify significant differences. The obtained data were analyzed with SPSS 23 package program. In this study, the principal component analysis (PCA) was used to determine the correlation between treatments and traits. PCA biplot graph were prepared using the Comprehensive R Archive Network [28].

## 3. Results and Discussion

### 3.1. Fruit Properties

**Figure 3:**
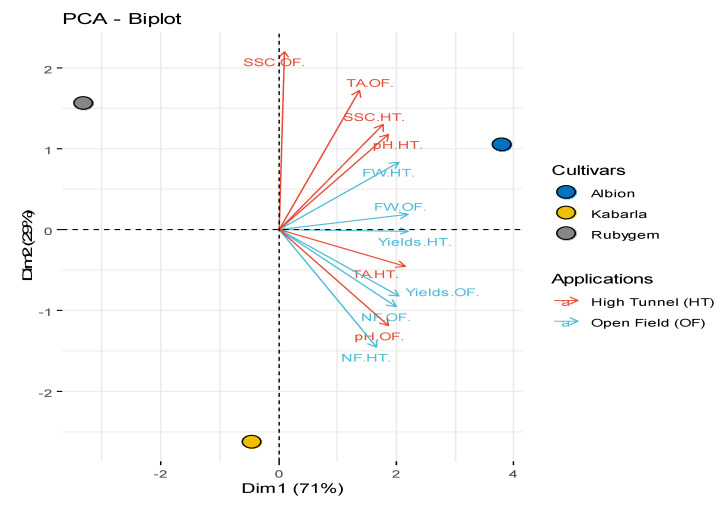
Principal component analysis (PCA) for fruit traits of different varieties and different applications. ^SSC^: Soluble solid content, ^TA^: Acidity, ^FW^: Fruit weight, ^NF^: Fruit number.

In this study, principal component analysis (PCA) was performed to determine the correlation between different growing conditions and agromorphological properties and biochemical contents of strawberry fruits (Figure 3). According to PCA analysis, strawberry varieties differed statistically from each other in terms of agromorphological characteristics. Rubygem was located in the first region, Albion in the second region and Kabarla in the third region in the PCA plot. It was observed that Albion cultivar was superior to other cultivars in terms of agromorphological characteristics under high tunnel and open field cultivation conditions. A negative correlation was determined between TA (Titratable cidity) and pH in both growing conditions. It has been observed that there is a positive correlation between SSC, pH and FW (Fruit Weight) in aquaculture under high tunnel conditions. In terms of fruit characteristics, statistical differences were found between applications and cultivars (*p* < 0.05). When the strawberry varieties included in the study were examined, the highest yield was obtained from Albion (542.743 g plant^−1^ and 486.163 g plant^−1^, respectively) in both high tunnel application and open field application. In the evaluation made in terms of different cultivation methods, it was seen that the high tunnel application had a positive effect on the amount of yield. As seen in Table 1, fruit weight was determined between 8.800–14.927 g fruit^−1^. The highest fruit weight was determined as 14.927 g fruit^−1^ in Albion variety grown in high tunnel application. While there was no difference in the number of fruits between applications and varieties, the highest fruit number was determined as 41.667 per plant in Albion grown in open field conditions (*p* < 0.05). There was no difference in SSC between applications and varieties. SSC was measured between 8.476% and 9.973%. In terms of pH content, no statistically significant difference was detected when the cultivations in open field and high tunnel were compared with each other. The titratable acid content in the fruits was determined between 0.780% and 1.047% (Table 1).

According to the results of different studies, differences were observed among strawberry varieties in terms of parameters such as fruit yield, fruit number and fruit size [29,30,31]. With the use of different growing systems such as glass greenhouse or high plastic tunnel, earliness can be possible in addition to increasing fruit yield and quality in strawberry, although it varies between applications and varieties [32,33]. As stated in different strawberry varieties [34], the soluble solid content of the fruit increases with ripening, while it tends to decrease in contrast to the titratable acidity ratio [35]. The pH, which has a positive effect on sensory properties in fruit, also increases with ripening [35,36]. Gündüz and Özdemir [33] stated that strawberry varieties show variability in terms of yield, fruit characteristics and earliness in greenhouse applications, especially in plastic greenhouse applications. In a study using different strawberry varieties, the average fruit yield, fruit number and water-soluble dry matter content of the varieties grown in the low plastic tunnel were found to be higher than the open field [37]. In another study, Kabarla was the variety with the highest yield among strawberry varieties [38]. Kuru Berk [39], determined the yield amount, fruit weight, soluble solid content, pH and titratable acid amount in Kabarla and Albion varieties and determined that there were differences in these parameters between applications and varieties. In this study, as in the others, differences were observed between the applications and varieties in terms of the results achieved.

### 3.2. Organic Acids

**Figure 4:**
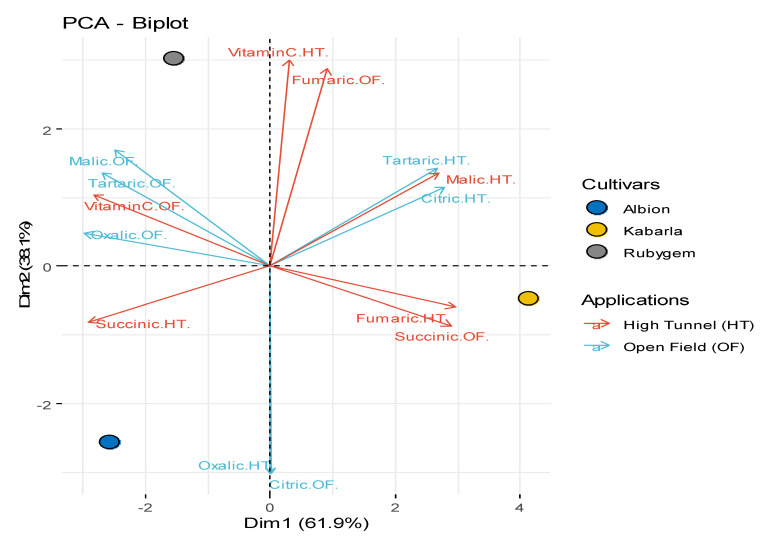
Principal component analysis (PCA) for organic acids of different varieties and different applications.

Oxalic acid, tartaric acid, citric acid, malic acid, succinic acid, fumaric acid and vitamin C contents were determined in the fruits of three strawberry varieties used in this study, and the results are presented in Table 2. According to the principal component analysis, it was observed that high tunnel and open field cultivation had a significant effect on the organic acid content of strawberry varieties (Figure 4). Looking at the correlation between organic acids, it was determined that there was a negative correlation between succinic acid and malic acid, tartaric acid, and citric acid. A parallel correlation was observed between vitamin C, malic acid, tartaric acid, and oxalic acid. A negative correlation was found between succinic acid and vitamin C. When the data were examined, statistically significant differences emerged between the applications and varieties in terms of organic acid content (*p* < 0.05). Among the determined organic acids, malic acid (427.570 mg 100 g^−1^ and 870.729 mg 100 g^−1^) was found to be the highest and dominant organic acid in the fruits of all strawberry varieties. Organic acids in fruits also affect different physiological changes, especially taste formation. In accordance with this study, the malic acid content was found to be high in different studies [12,40]. Kabarla variety had the highest malic acid content in high plastic tunnel application, while it was the variety with the lowest malic acid content in open field application. In addition, vitamin C content varied between 14.195 and 25.419 mg 100 g^−1^, and the highest value was determined in Rubygem in high tunnel and open field cultivation.

Perez et al. [41] found higher citric acid content in their study. High amounts of oxalic, citric, and succinic acids were found in strawberry cultivars in both cultivations. Consistent with these results, fumaric acid was reported as the least abundant organic acid in strawberries [42]. The findings of this study are mostly in line with other research showing the organic acid richness of fruits. Differences in studies may be related to the species, varieties, practices, environmental conditions, and genetic factors examined [43,44]. During fruit ripening, citric acid tends to decrease, malic acid irregular and ascorbic acid tend to increase [35]. In a study using 10 strawberry varieties; Organic acids were tried to be determined in strawberry fruits and citric acid and malic acid were reported as dominant organic acids, although they differ between varieties [4]. While citric acid and malic acid were determined as dominant organic acids in Kabarla strawberry variety, citric acid, malic acid, and succinic acid were determined as dominant organic acids in Albion strawberry variety [39]. Consistent with these studies, in our study, the fumaric acid content was the lowest level of organic acid in all cultivars. Organic acids and sugars are the main factors affecting the sensory properties of fruits. The organic acid-sugar ratio is also an important criterion to characterize fruit flavor. Organic acids in the fruit are effective in taste and aroma, not only in fresh consumption but also as a processed product. For example, significant amounts of malic acid, citric acid and oxalic acid contents were determined in strawberry jam made from fruit puree without additives [45]. Organic acids show antioxidant properties, which explains their widespread use for pharmacological purposes. The organic acids, which has a decisive effect on the taste of strawberry fruit, varies according to genotype and variety [46,47]. The results showed that high tunnel conditions had little effect on the amount of phenolic compounds and organic acids in strawberry cultivars. In a study, it was reported that the antioxidant capacity of the fruits of plants grown in open field was higher than those in plastic tunnel conditions [48].

### 3.3. Phenolic Compounds

**Figure 5:**
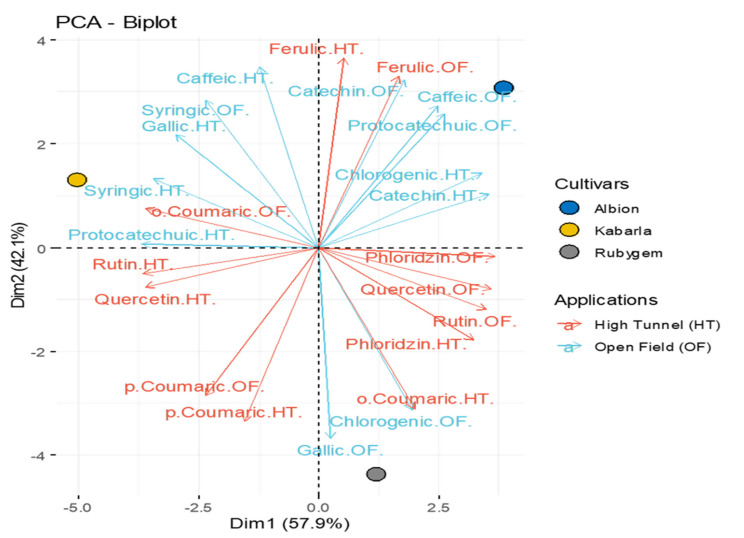
Principal component analysis (PCA) for phenolic compounds of different varieties and different applications.

In this study, there were significant differences in the phenolic contents of strawberry fruits according to different growing conditions. According to Principal component analysis, Kabarla, Albion and Rubygem varieties were located in the first, second and fourth regions, respectively, and the varieties differed from each other in terms of phenolic compounds (Figure 5). Gallic acid was found to be the highest compound among phenolic acids. It was observed that there was a negative correlation between gallic acid and ferulic acid. Among the varieties grown in open field, the highest gallic acid content was determined in Rubygem variety and the highest values in high tunnel cultivation were determined in Kabarla variety. While a positive correlation was found between quercetin and rutin in high tunnel cultivation, a negative correlation was found between these two compounds and catechin and chlorogenic acid. In this study, gallic acid content was determined between 9.246 and 31.680 mg 100 g^−1^, while protocatechuic acid content was found between 2.602 and 9.948 mg 100 g^−1^. Catechin and chlorogenic acid contents were determined in the range of 2.135–7.925 mg 100 g^−1^ and 5.995–10.146 mg 100 g^−1^, respectively (Table 3). Phenolic compounds are secondary plant metabolites with strong antioxidant activities that are common in all plants, especially in fruits and vegetables. Especially fruits such as blueberry, currant, blackberry, strawberry, and raspberry, which are red-dark colored fruits, are also rich food sources of phenolic compounds and antioxidants [49,50,51]. Phenolic compounds are known as important antioxidant compounds in strawberries [52]. The most important reason for the increase in studies on phenolic compounds in recent years is that the consumption of fruits containing these compounds has a high level of protective properties against diseases that cause oxidative damage such as heart disease, stroke, and cancer [53].

In this study, statistically significant differences were determined between the applications and the phenolic acid contents of the genotypes (*p* < 0.05). These differences between varieties and applications have also been reported in other studies [54,55,56,57,58,59,60,61,62,63,64,65]. Different studies have been conducted to identify and determine phenolic compounds in strawberry fruits [17]. Greenhouse applications give different results in different strawberry varieties and increase the total amount of phenolic compounds positively [7]. It has been stated that the differences between applications and varieties in terms of the amount and composition of phenolic compounds may be due to factors such as genotype, environmental conditions, growing techniques and storage conditions, as observed in berry fruits [18,66]. In a study conducted in Ecuador, different fruit species from the Rosaceae family and some phenolic compounds and their derivatives were determined in different amounts in strawberry fruits [67]. It is known that phenolic compounds strongly affect fruit quality and contribute to both sensory properties and nutritional values. Anthocyanins are quantitatively the most important type of polyphenol in strawberries. Anthocyanins are one of the phenolic compounds, responsible for the bright red color of the fruit. The concentration and composition of anthocyanins are important for the sensory quality of fruits and products, in addition to their possible health benefits. Recent studies have focused on identifying the bioactive compound of various strawberry cultivars and revealing the best and identifying factors that influence the composition of this unique fruit [68,69]. Phenolic compounds are chemicals that have important contributions to human health. In terms of these compounds, which are strong antioxidants and whose concentration varies depending on ripening, it has been shown in the study that Albion strawberry variety is a good source of phenolic compounds [35]. Urün et al. [4] reported that ellagic acid was the predominant phenolic compound among phenolic compounds in 10 strawberry cultivars. As seen in this study, phenolic acid contents varied in terms of applications and varieties. In this study, gallic acid, chlorogenic acid, catechin and protocatechuic acid contents were found to be more dominant than other phenolics. Similarly, catechin and gallic acid were dominant phenolic compounds as a result of putrescine and mycorrhiza applications in Albion and Kabarla strawberry cultivars [39].

## 4. Conclusions

In order to preserve the fruit quality characteristics in fresh or industrial consumption, besides choosing high quality strawberry varieties, it is necessary to establish and use different growing systems. In this way, strawberry varieties with high quality and high yield will be grown in a long season regardless of ecological conditions, and it will be possible to find both fresh and industrial products. In addition to fruit yield, chemical content is also important. As a result of the increase in awareness of healthy nutrition, the phytochemical properties of strawberry with scientific studies and the determination of the disease-preventing properties of these substances, the demand for strawberries has increased gradually. In this study, the effect of open field and high tunnel applications on fruit yield and fruit quality characteristics of three commonly grown strawberry cultivars were investigated and their phytochemical properties and their importance for human health were determined by using advanced analysis techniques. Thanks to the determination of many biochemicals such as phenolic compounds and organic acids, which have important contributions to nutrition and health, the benefits of strawberry are better understood and the demand for this fruit species is increasing. According to the results obtained, fruit characteristics, phenolic compounds and organic acid contents differed in terms of varieties and applications. Fruit yield was high in Albion variety in both applications.

In this study, gallic acid, protocatechuic acid, catechin and chlorogenic acid were found to be higher than other phenolic compounds. Among the organic acids determined in strawberry fruits, malic acid and oxalic acid had the greatest values in high tunnel application. It turns out that the data obtained in terms of investigating important phytochemicals in strawberry, which is a functional product evaluated in the food industry as well as fresh consumption, is compatible with other studies. Although earliness is a known fact in high tunnel cultivation, it has been observed that there is limited information on how organic acids and phenolic compounds change. Therefore, it is thought that the obtained results are important in terms of providing reference for future studies.

## Figures and Tables

**Table 1:** Fruit properties of strawberry cultivars grown in open field and high plastic tunnel.

Applications	Varieties	Yield (g/Plant)	Fruit Weight (g/Fruit)	Fruit Number (Number/Plant)	SSC (%)	pH	Acidity (%)
Open Field	Albion	486.163 ± 19.60 a	11.470 ± 0.73 ab	41.667 ± 5.69 a	8.830 ± 0.52 a	3.793 ± 0.27 a	0.916 ± 0.02 ab
	Kabarla	407.570 ± 12.98 ab	10.787 ± 0.36 ab	37.676 ± 2.40 a	8.476 ± 0.57 a	3.776 ± 0.32 a	0.867 ± 0.05 ab
	Rubygem	176.285 ± 13.20 b	13.010 ± 1.24 ab	14.500 ± 3.50 b	8.705 ± 0.96 a	3.740 ± 0.15 a	0.780 ± 0.03 b
High Plastic Tunnel	Albion	542.743 ± 12.87 a	14.927 ± 1.08 a	36.641 ± 3.71 a	9.973 ± 0.56 a	4.133 ± 0.15 a	1.047 ± 0.06 a
	Kabarla	376.920 ± 10.93 ab	10.060 ± 0.39 ab	37.566 ± 2.60 a	8.946 ± 0.74 a	3.763 ± 0.05 a	0.903 ± 0.05 ab
	Rubygem	244.885 ± 13.10 b	8.800 ± 3.54 b	29.000 ± 4.26 ab	9.180 ± 0.23 a	3.740 ± 0.18 a	0.938 ± 0.05 ab

Difference between means represented with the different letter in the same column is significant at 0.05 level.

**Table 2:** The content of organic acids in fruits of strawberry cultivars grown in open field and high plastic tunnel (mg/100 g).

Applications	Varieties	Oxalic Acid	Tartaric Acid	Citric Acid	Malic Acid	Succinic Acid	Fumaric Acid	Vitamin C
Open Field	Albion	107.774 ± 09.15 c	41.845 ± 3.96 ab	182.249 ± 1.17 a	594.166 ± 4.21 d	152.410 ± 6.83 cd	2.408 ± 0.01 d	23.978 ± 0.83 a
	Kabarla	39.634 ± 1.61 d	10.116 ± 0.10 b	143.783 ± 3.41 ab	427.570 ± 5.24 e	278.015 ± 3.16 b	9.107 ± 0.37 c	18.763 ± 0.49 b
	Rubygem	145.373 ± 4.96 b	57.062 ± 0.01 a	79.090 ± 1.05 b	723.726 ± 0.05 c	129.038 ± 0.43 d	12.842 ± 0.09 b	25.419 ± 0.79 a
High Plastic Tunnel	Albion	211.959 ± 6.59 a	16.649 ± 0.65 b	75.191 ± 4.97 b	749.491 ± 2.19 bc	397.680 ± 6.95 a	13.781 ± 0.08 b	14.195 ± 0.08 c
	Kabarla	151.002 ± 4.34 b	69.296 ± 1.32 a	222.372 ± 4.24 a	870.729 ± 5.11 a	161.822 ± 2.48 c	18.756 ± 0.30 a	19.318 ± 0.31 b
	Rubygem	48.789 ± 1.09 d	48.117 ± 6.25 ab	150.222 ± 2.34 ab	819.426 ± 8.62 ab	301.133 ± 0.28 b	13.452 ± 0.33 b	25.334 ± 0.72 a

Difference between means represented with the different letter in the same column is significant at 0.05 level.

**Table 3:** (a). The content of phenolic compounds (mg/100 g) in fruits of strawberry cultivars grown in open field and high plastic tunnel (b). The content of phenolic compounds (mg/100 g) in fruits of strawberry cultivars grown in open field and high plastic tunnel.

(a)							
Applications	Cultivars	Gallic acid	Protocatechuic Acid	Catechin	Chlorogenic Acid	Caffeic Acid	Syringic Acid
Open Field	Albion	19.079 ± 0.35 bc	7.890 ± 0.26 ab	6.423 ± 2.85 a	6.261 ± 0.43 a	2.094 ± 0.06 a	0.303 ± 0.19 b
	Kabarla	21.244 ± 0.95 bc	2.746 ± 0.03 d	3.493 ± 0.03 a	5.995 ± 0.30 a	1.157 ± 0.02 b	0.494 ± 0.08 ab
	Rubygem	31.680 ± 0.41 a	2.602 ± 0.48 d	2.135 ± 0.58 a	6.803 ± 2.62 a	1.064 ± 0.01 bc	0.068 ± 0.04 b
High Plastic Tunnel	Albion	14.699 ± 1.45 cd	3.883 ± 0.50 cd	7.925 ± 0.27 a	10.146 ± 0.72 a	0.974 ± 0.12 bc	0.202 ± 0.02 b
	Kabarla	25.396 ± 2.57 ab	9.948 ± 0.94 a	3.495 ± 0.37 a	6.869 ± 0.69 a	1.025 ± 0.10 bc	1.030 ± 0.10 a
	Rubygem	9.246 ± 0.51 d	5.604 ± 0.21 bc	5.513 ± 2.35 a	8.015 ± 0.08 a	0.599 ± 0.15 c	0.129 ± 0.01 b
**(b)**							
**Applications**	**Cultivars**	**Ferulic acid**	***p*-Coumaric acid**	**o-Coumaric acid**	**Rutin**	**Phloridzin**	**Quercetin**
Open Field	Albion	1.523 ± 0.06 a	0.522 ± 0.07 d	0.251 ± 0.07 c	0.521 ± 0.01 c	0.962 ± 0.05 a	0.949 ± 0.01 a
	Kabarla	0.638 ± 0.02 a	1.329 ± 0.05 bc	0.735 ± 0.02 ab	0.355 ± 0.01 c	0.327 ± 0.07 a	0.193 ± 0.01 c
	Rubygem	0.163 ± 0.04 a	1.462 ± 0.01 bc	0.299 ± 0.03 c	0.528 ± 0.01 c	0.801 ± 0.02 a	0.887 ± 0.08 a
High Plastic Tunnel	Albion	1.271 ± 0.29 a	1.116 ± 0.07 c	0.477 ± 0.01 bc	0.798 ± 0.01 bc	0.681 ± 0.02 a	0.363 ± 0.18 bc
	Kabarla	1.082 ± 0.62 a	1.818 ± 0.15 ab	0.274 ± 0.08 c	1.507 ± 0.13 a	0.400 ± 0.23 a	0.791 ± 0.04 ab
	Rubygem	0.753 ± 0.03 a	2.248 ± 0.11 a	0.871 ± 0.11 a	1.096 ± 0.14 ab	0.760 ± 0.18 a	0.569 ± 0.07 abc

Difference between means represented with the different letter in the same column is significant at 0.05 level.

## Data Availability

All-new research data were presented in this contribution.
